# Molecular Insights into the MAPK Cascade during Viral Infection: Potential Crosstalk between HCQ and HCQ Analogues

**DOI:** 10.1155/2020/8827752

**Published:** 2020-12-31

**Authors:** Tapan Kumar Mohanta, Nanaocha Sharma, Pietro Arina, Paola Defilippi

**Affiliations:** ^1^Natural and Medical Sciences Research Center, University of Nizwa, Nizwa 616, Oman; ^2^Institute of Bioresources and Sustainable Development (IBSD), Imphal 795001, India; ^3^UCL Division of Medicine, Bloomsbury Institute for Intensive Care Medicine, London, WC1E 6BT, UK; ^4^Department of Molecular Biotechnology and Health Sciences, University of Turin, Turin 10126, Italy

## Abstract

The mitogen-activated protein kinase (MAPK) pathway links the cell-surface receptors to the transcription machinery, transducing the extracellular signals into several outputs, which may also adapt the host defense mechanism to viral attacks. The Severe Acute Respiratory Syndrome CoronaVirus 2 (SARS-CoV-2) that causes the COrona VIrus Disease 2019 (COVID-19) has infected upwards of nearly 70 million people and worldwide has claimed more than 1,600,000 deaths. So far, there continues to be no specific treatment for this novel coronavirus-induced disease. In the search to control the global COVID-19 pandemic, some eastern and developing countries have approved a variety of treatments with controversial efficacy, among which is the use of the antimalarial hydroxychloroquine (HCQ). Interestingly, prior data had indicated that the HCQ/CQ could influence the MAPK cascade. The main aim of this review is to address molecular mechanisms, beyond drugs, that can be helpful against viral infection for this and future pandemics. We will highlight (1) the contribution of the MAPK cascade in viral infection and (2) the possible use of MAPK inhibitors in curbing viral infections, alone or in combination with HCQ and quinoline analogues. We are convinced that understanding the molecular patterns of viral infections will be critical for new therapeutical approaches to control this and other severe diseases.

## 1. Introduction

**Figure 1:**
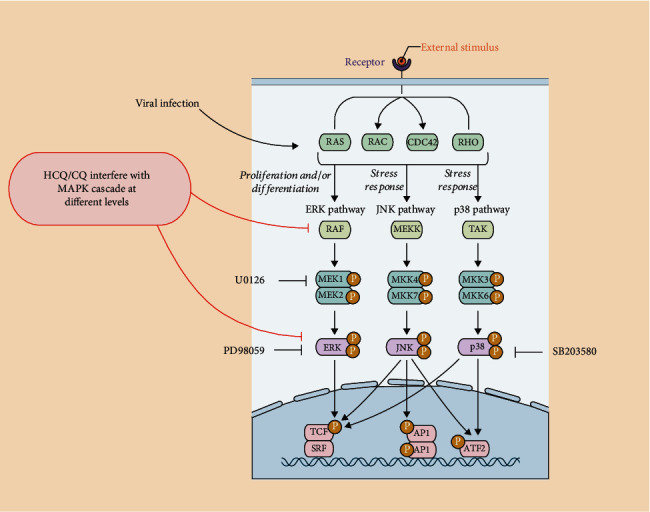
The MAPK pathway: activators and inhibitors. This picture summarizes the main components of the MAPK pathways. Upon membrane receptor activation, or viral infection [3], Ras family GTPases trigger signaling cascades, leading to the activation of ERK, JNK, and p38, which in turn regulate transcription of a plethora of genes involved in many cell functions, including regulation of viral infection/replication. Inhibitors of specific MAPK proteins, discussed in this review, are shown: U0126 for MEK1, SB203580 for p38, and PD 98059 for ERK1/2. As discussed in the text, CQ and HCQ have the ability to interfere with the pathway at the level of Raf or, downstream, on ERK1/2 and p38 activation.

The mitogen-activated protein kinases (MAPKs) are a family of highly conserved serine-threonine protein kinases that connect extracellular cues, including viral infections, to the cell nucleus and thereafter into various outputs, which may also affect the mechanism of host defense and apoptosis (Figure 1). The first modules in the signaling cascade are the small GTPases, including Ras, which are triggered by the canonical receptor tyrosine kinase- (RTK-) Grb2-SOS signaling scheme or by viral infection [1–3]. As shown in Figure 1, the Ras family of proteins, including members like the K-Ras, H-Ras, and N-Ras, primarily participates in transmitting the extracellular signals into the cells [4]. Ras activates the Raf-1 kinase, which, in turn, phosphorylates and activates MEK (MEK1 and MEK2). MEK phosphorylates and activates a mitogen-activated protein kinase (MAPK). The MAPK cascade includes the Extracellular Signal-Regulated Kinase (ERK1/2), the c-Jun NH2-terminal kinase (JNK), and p38 [1]. Over the past several decades, intense work is in progress to develop and evaluate compounds that can target the MAPK pathway components to treat inflammatory and neurodegenerative diseases and cancer [5–7].

**Figure 2:**
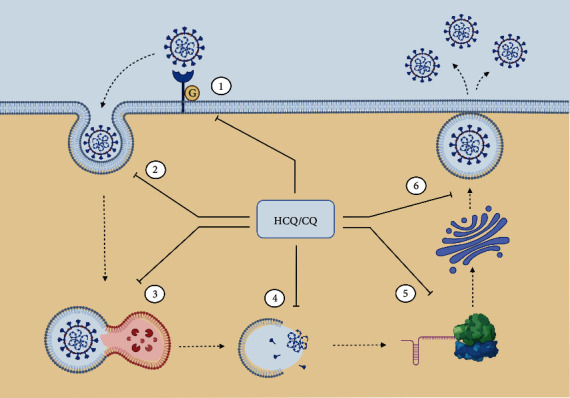
Summary of the possible antiviral properties of CQ, HCQ, and analogues on the SARS-CoV-2 replication cycle. The broad antiviral spectrum of CQ, HCQ, and analogues may depend on their ability to interfere with (1) the early phases of viral infection, mediated by ACE2 and TMPRSS2, affecting the viral particles binding to their receptors—this occurs interfering with the biosynthesis of sialic acid [33] and with the glycosylation of either the spike protein itself [35] or the cell surface receptor ACE2 [36]; (2) the pH-dependent endocytosis of viral particles, by increasing the endosomal pH; (3) the low pH-dependent endosome-mediated viral entry of viruses and the lysosomal-dependent disruption of the viral particles; (4) the viral particle uncoating and release of nucleic acid and enzymes necessary for its replication; (5) the posttranslational modification of viral proteins, by blocking the low pH-dependent action of proteases and glycosyltransferases on viral envelope glycoproteins, within the trans-Golgi network vesicles; (6) the viral budding preventing the release of viral particles.

Severe Acute Respiratory Syndrome CoronaVirus 2- (SARS-CoV-2-) mediated COVID19 is a global pandemic that has affected nearly 70 million individuals, claiming more than 1,600,000 lives across the world. SARS-CoV-2 is a single-stranded RNA-enveloped virus (reviewed in [8]), with a 29,881 bp long RNA, encoding the structural proteins S, E, M, and N and the nonstructural proteins, 3-chymotrypsin-like protease, papain-like protease, and RNA-dependent RNA polymerase. As shown in Figure 2, viral cell entry is mediated by the binding of the cell-surface glycosylated S proteins to the host cell receptor angiotensin-converting enzyme 2 (ACE2), and endocytosis is promoted by the activation of the S protein by the TM protease serine 2 (TMPRSS2), located on the host cell membrane. Into the cell, the released viral RNA is translated into the proteins while replication and transcription of the viral RNA genome occur via protein cleavage and assembly of the replicase-transcriptase complex. Replicated viral RNA and structural proteins are synthesized, assembled, and packaged in the host cell, before viral particle release.

As of late fall 2020, no specific treatment for the novel coronavirus-induced disease has been found. During the global crisis, such as the one caused by COVID-19, there is an urgent need to discover new drugs or to reposition old medicines for new uses. The World Health Organization (WHO) recently recommended against Remdesivir (a nucleotide analogue prodrug with antiviral activity against hepatitis B virus and human immunodeficiency virus), suggesting no important effect on mortality for COVID-19, need for mechanical ventilation, time to clinical improvement, and other patient-important outcomes (WHO, https://www.who.int/news-room/feature-stories/detail/who-recommends-against-the-use-of-remdesivir-in-covid-19-patients). Moreover, a systematic review of randomized trials and observational studies addressing the efficacy of the humanized monoclonal antibody anti-interleukin- (IL-) 6-receptor tocilizumab showed that it may reduce the risk of mechanical ventilation in hospitalized COVID-19 patients, but not short-term mortality [9].

CQ (N4-(7-chloro-4-quinolinyl)-N1,N1-diethyl-1,4-pentanediamine) has long been employed in the treatment of malaria and amebiasis. Hydroxychloroquine (HCQ) sulfate is one of its derivatives, shown to exert at least 40% less toxicity than CQ in animals [10]. It continues to be widely used to treat autoimmune diseases, such as systemic lupus erythematosus and rheumatoid arthritis. Initially, HCQ was seen to potentially modulate COVID-19 disease [11, 12] suggesting its prophylactic usage against the coronavirus. Recently, mefloquine, a quinoline derivative of the same antimalarial class of agents that includes CQ and HCQ, came out from a large drug screening as a promising molecule that can impair in vitro viral replication [13]. However, to date, no data are available to support that HCQ exerts a favorable effect on the outcomes in COVID-19 patients, mainly because of the absence of a commonly agreed dosing protocol based on pharmacokinetic studies, with dose and treatment duration that, at present, vary across national guidelines and clinical study protocols [14]. Noteworthy, a very recent review underlines that HCQ is consistently effective against COVID-19 when provided early in the outpatient setting; it is overall effective against COVID-19, it has not produced a worsening of the disease, and it is safe [15]. Interestingly, the authors of this systematic review show all the available details of 43 studies around the world showing (i) positive results with HCQ patients (20,441 COVID-19 patients), (ii) no improvements (11,209 COVID-19 patients), or (iii) worse results (7,679 COVID-19 patients). They indicate that 53% of the studies showed a definitive positive effect of HCQ vs. COVID-19, with significant biases on studies showing worst outcome [15]. Overall, the guideline development groups recognized that basic and clinical research is still needed, especially to provide additional molecular mechanisms and higher certainty of evidence for specific groups of patients. Thus, detailed molecular and functional studies on HCQ and its derivatives are highly necessary in the context of COVID-19 infection by *in vitro* and *in vivo* assays. Therefore, the objective of this short review is to highlight the available data on the impact of the MAPK cascade on the viral infection and whether and how MAPK inhibitors and HCQ or analogues can inhibit it. In particular, we wish to address two issues: (i) review the possible involvement of the MAPK cascade in SARS-Cov-2 infection; (ii) highlight a putative cross talk between inhibition of the MAPK signaling and the treatment with HCQ/CQ. Basic molecular studies merit further investigation, to discover new strategies to control the replication of several viruses, with combinatorial approaches targeting the MAPK cascade, together with the HCQ derivatives as a new generation of antivirals.

## 2. The MAPK Cascade in Viral Infections

It is already well recognized that the MAPK pathway (Figure 1) may be activated in viral infections [3], as viruses are ultimately dependent upon the host cell for their life cycle. Cellular signaling pathways are exploited by viruses for their own effective replication, translation, transport across the nuclear membrane, capsid assembly, and spreading, as well as reactivation of virus latency [3]. Of interest, the MAPK cascade also participates in regulating the immune response [16] and apoptosis [17] in virus-infected cells. A variety of DNA and RNA viruses induces signaling through the MAP kinase cascades in infected host cells [3, 18, 19]. The MAPK signaling may act either as a positive or negative regulator of viral replication, exploiting the exogenous MAPK pathway activators, such as G-protein-linked or tyrosine kinase receptors, to promote their own replication [20]. The MAPK pathways can be active on contact with both the live or inactivated virus cells [20], and even the viral secretory proteins can trigger the ERK1/2 activation, possibly because of the homology of the viral proteins with the Epidermal Growth Factor (EGF) and Transforming Growth Factor (TGF) [21]. Host factors may either support (proviral effect) or inhibit (antiviral effect) the viral replication. The proviral factors may act as targets in the development of antiviral therapeutics [3]. Indeed, such extensive use of the MAPK cascade by viruses implies that this pathway may be a good target for developing broad-spectrum antiviral drugs. Several viruses can be sensitive to the MAPK kinase inhibitor, which may possess the potential to behave as antiviral agents, as summarized in [3] (see also Figure 1). Interestingly, drugs targeting the p38 MAPK inhibitor, SB203580, inhibited the effective phosphorylation of HSP-27, CREB, and eIF4E in SARS-CoV-infected cells, showing promise as a new class of antiviral agents [22].

Influenza A viruses, a relevant human global pathogen group, induces a biphasic activation of the Raf/MEK/ERK1/2 cascade that can be inhibited by treatment with the MEK inhibitor U0126. This inhibitor induces the nuclear retention of the viral ribonucleoprotein complexes (RNPs), with impaired function of the nuclear export protein (NEP/NS2), and concomitant inhibition of virus production. Generally, signaling through the MAPK pathway may be crucial in virus production, and RNPs export from the nucleus during the viral life cycle [23]. The U0126 inhibitor also interferes in the spread of the Borna Disease Virus (BDV) to the neighboring cells, influencing the BDV-host cell interaction [24]. The MAPKs, including ERK1/2, JNK, and p38, greatly influence the infection of the coronaviruses, such as mouse hepatitis virus and SARS-CoV [25, 26], while the ER stress caused by the Japanese Encephalitis virus infection activates the p38 mitogen-activated protein kinase (MAPK) and host cell apoptosis [27].

Moreover, inhibition of the ERK1/2 pathway is also related to a decline in the viral progeny of varicella zoster [28]. The ERK1/2 cascade is partially activated during the varicella zoster viral infection and contributes towards inducing cell survival signals. For instance, the c-Raf is inactive, whereas its downstream kinases MEK1/2 and ERK1/2 are transiently phosphorylated. Inhibition of MEK1/2 and ERK1/2 signaling impairs the virus replication and increases the apoptotic response, by suppressing the phosphorylation of BAD, a cell death regulator and an indirect cytosolic target of the ERK1/2 [28]. New insights into the molecular basis of viral hepatitis reveal that three of these agents—hepatitis B, C, and E viruses (HBV, HCV, and HEV, respectively)—modulate the MAPK signaling pathway [29]. Dysregulation of the signaling mechanisms such as the ERK1/2 activation should promote several different stages of the HBV infection. Among them, the HBxAg protein does multiple functions, including signal transduction, transcriptional activation, DNA repair, and inhibition of protein degradation. The HBxAg directly stimulates the ERK1/2 pathway by activating Ras, leading to cell progression into the S phase through cyclin D1 upregulation [29]. Remarkably, the inhibition of the Raf-associated MEK-ERK1/2 reduces the reactivation of Kaposi's sarcoma-associated herpes virus [30–32], reviewed in [33]. Overall, these data open up new avenues in terms of developing antiviral drugs.

## 3. Possible Effects of CQ, HCQ, and Derivatives on SARS-CoV-2 Replication Cycle

CQ, HCQ, and derivatives can act at different steps of the viral cycle (Figure 2). They can impair the preentry step by interfering with the biosynthesis of sialic acid, which is a critical component of the cell surface receptor ligand for the virus recognition [34]. This mechanism could explain the broad antiviral spectrum of those drugs since both the human coronavirus HCoV-O43 and the orthomyxoviruses use sialic acid moieties as receptors [35]. CQ and HCQ could inhibit the SARS-CoV-2 entry by altering the glycosylation of the spike protein itself [36], as also suggested for the HCQ analogues. Besides, CQ and HCQ impair in vitro the terminal glycosylation of ACE2 without significant change of cell surface ACE2 and, therefore, might be potent inhibitors of SARS-CoV-2 infections [37]. Indeed, very recently, HCQ has been proven to be effective as an aerosol to reduce and even prevent severe clinical symptoms after SARS-CoV-2 infection [38]. Viral entry involves endocytosis, and during this process, an activation step in endosomes at acidic pH leads to fusion of the viral and endosomal membranes, with consequent release of the viral SARS-CoV-1 genome into the cytosol [39]. Both CQ and HCQ increase the endosomal pH, thus impairing the low pH-dependent virus-endosome fusion and endosome-mediated viral entry of viruses such as the chikungunya virus [40] and mouse hepatitis coronavirus [41]. The lysosomal-dependent disruption of the viral particles, with uncoating, release of nuclei acid, and enzymes necessary for its replication, is thus impaired. CQ can also interfere with the posttranslational modification of viral proteins. The low pH is indeed required for the action of proteases and glycosyltransferases on viral envelope glycoproteins, within the endoplasmic reticulum or the trans-Golgi network vesicles, possibly preventing the release of the viral genome or leading to less infectious neosynthesised HIV virus particles [42]. In the herpes simplex virus (HSV) model, CQ impairs budding with accumulation of noninfectious HSV-1 particles in the trans-Golgi network PMID: 6492263. Recently, a trans-Golgi network localization signal had been found on the C-terminal domain of the MERS-CoV M protein [43]. Moreover, the CQ impairs autophagy by interfering with the fusion of the autophagosome with the lysosomes rather than influencing the acidity and/or degradative activity of this organelle [44].

## 4. Immunopathogenesis of Severe COVID-19

**Figure 3:**
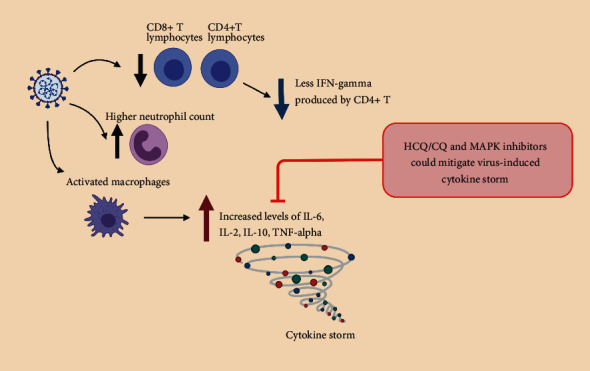
Immunomodulation by Sars-CoV-2 infection and potential inhibitors. In COVID-19 patients, as elegantly reviewed in [45, 46, 48], SARS-Cov-2 infection causes a highly generalized proinflammatory state, the so-called “cytokine storm” by activated macrophages that produce proinflammatory cytokines, such as IL-6, IL-2, IL-10, and TNF-alpha, with a reduction in IFN-gamma production by CD4+ T cells. In parallel, a higher neutrophil count, a lower number of lymphocytes, and lymphopenia are observed. HCQ/CQ have been shown to downregulate cytokine production in different diseases [49], acting on the MAPK cascade in human epithelial cells upon coronavirus infection [54], thus contributing to impair the cytokine storm.

Poorer outcomes in COVID-19 patients had been correlated with a high generalized proinflammatory state, the so-called “cytokine storm” that follows the rapid viral replication in the early phases of SARS-Cov-2 infection, as recently reviewed in [45, 46] (Figure 3). Proinflammatory cytokines are predominantly produced by activated macrophages and are involved in the upregulation of inflammatory reactions. SARS-CoV-2 infection induces an increase in proinflammatory cytokines, such as IL-6, IL-2, IL-10, and TNF-alpha. In COVID-19 patients, this correlates with a higher neutrophil count, a lower number of lymphocytes, and lymphopenia. Interestingly, disease severity correlates with a decrease in CD8+ T cells, where CD8+ T cells are the main inflammatory cells and play a vital role in virus clearance, as well as in CD4+, with a reduction in IFN-gamma production by CD4+ T cells. Indeed, this is in line with the data obtained from SARS-CoV-1, where viral replication produces proteins that can block antiviral interferon (IFN) responses [47]. Moreover, excessive infiltration of proinflammatory cells, mainly involving macrophages and T-helper 17 cells, has been found in lung tissues of patients with COVID-19 by postmortem examination. The attenuated and delayed IFN responses provoke an accumulation of pathogenic inflammatory macrophages. This, in turn, results in an even higher production of cytokines. This cytokine storm produces an excessive inflammatory and immune response, especially in the lungs, leading to acute respiratory distress syndrome (ARDS), pulmonary edema, apoptosis of epithelial cells, vascular damage, and multiorgan failure [48].

The current hypothesis is that HCQ and derivatives could mitigate the severe progression of COVID-19, inhibiting the cytokine storm. This hypothesis has been elegantly described in a recent review [49], where a large amount of literature describes that CQ and HCQ can inhibit the production of IL-1, IL-2, IL-6, IL-17, and IL-22 in different disease conditions. In addition, CQ and HCQ interfere with the levels of IFN-alpha, IFN-gamma, and TNF-alpha. Moreover, CQ affects the stability between T-helper cell (Th)1 and Th2 cytokine secretion by augmenting IL-10 production in peripheral blood mononuclear cells (PBMCs). Similarly, HCQ also inhibits cytokine generation from all the B-cell subsets, where IgM memory B-cells exhibit the utmost cytokine production. Based on these data, CQ and HCQ are thus potential inhibitors of the COVID-19 cytokine storm.

## 5. Can CQ, HCQ, and Derivatives Affect MAPK Activation?

Further investigation into the earlier published literature regarding the mechanism of action of the HCQ revealed the possible impact on the MAPK pathway activation [50]. In different cell types, and at systemic levels, the CQ and derivatives can influence the activation of p38 MAPK and the cytokine release caused by the bacterial CpG DNA [51] as well as by the ERK1/2 [50] (Figure 1). The CQ reduced the ERK1/2 phosphorylation by blocking the phosphorylation event of MEK [50]. Interestingly, as already shown above, in these experiments, both CQ and PD98059, an ERK1/2 inhibitor, were effective in inhibiting human TNF-alpha transcription, thus indicating that both CQ and MAPK inhibitors could negatively downregulate the viral-induced cytokine storm. However, CQ appeared to mediate these effects by deactivating Raf, the upstream activator of MEK. These results were strengthened by functional data demonstrating that CQ and PD98059 interfered with TNF-alpha expression in several human and murine cell types. Consistent with these results, neither inhibitor blocked TNF-alpha production in murine RAW264.7 macrophages, a cell line that does not require MEK-ERK signaling for TNF production. Finally, they also showed that CQ could sensitize HeLa cells to undergo anti-Fas-mediated apoptosis, an effect observed when ERK1/2 activation is interrupted in this cell line [52]. Therefore, CQ rendered HeLa cells sensitive to anti-Fas treatment in a manner similar to PD98059. Overall, these data indicate that therapeutic concentrations of CQ interfere with ERK1/2 activation by a novel mechanism, an effect that could be responsible, at least in part, for the potent anti-inflammatory effects of this drug [50]. Indeed, the treatment with either CQ or the ERK1/2 inhibitor PD98059 causes the inhibition of the lipopolysaccharide- (LPS-) induced MAPK activation and TNF-alpha expression in the human THP-1 and murine AMJ2C-8 macrophage cell lines, but not in the RAW264.7 cells that do not require the MEK-ERK signaling for TNF-alpha production reviewed in [3, 50]. The CQ was also effective in inhibiting the p38 MAPK phosphorylation, c-Jun N-terminal kinase, and ERK1/2 together with a marked decrease of the lipopolysaccharide- (LPS-) induced IL-1 beta release in the human monocytic cell THP-1 cells [53]. Overall, these data show that CQ and derivatives could profoundly affect both the MAPK signaling and expression of the inflammatory cytokines in human epithelial and monocyte cells. Regarding human coronavirus 229E (HCoV-229E) infection of the human fetal lung cell line, L132, CQ significantly decreases the viral replication at concentrations lower than in clinical usage [54]. The CQ effect is dependent on the inhibition of the MAPKs involved in the replication of HCoV-229E. A correlation between the CQ used at concentrations lower than in clinical usage and the specific inhibition of the p38 MAPK has been described in HCoV-229E-infected L132 cells. Furthermore, the p38 MAPK inhibitor, SB203580, inhibits HCoV-229E viral replication [54]. Overall, these results indicate that the CQ, in low therapeutic concentrations, may interfere with the activation of the MAPK pathways at different levels, thus causing a downregulation of MAPK-dependent inflammatory pathways. These data strongly support the hypothesis that HCQ and derivatives could mitigate the severe progression of COVID-19, inhibiting the cytokine storm.

The complexity of the mechanisms of action of the MAPK cascade associated with the viral activation and progeny can be closely related to the mechanism of action of the CQ. As shown above, in the model of the HCoV-229 coronavirus, the CQ-induced virus inhibition occurs through the p38 MAPK inhibition [54]. Therefore, the HCQ treatment can exert a direct impact on the replication of the viral progeny through the inhibition of the MAPK pathway. Simultaneously, reactivation of the infection can also be inhibited by the HCQ through the inhibition of the ERK-MEK1/2 signaling cascade.

## 6. MAPK Activation in Severe COVID-19 Disease

The epidemiology of COVID-19 has not yet been fully understood. Estimates indicate that the number of positive people can be many times more than the numbers available at present, with countries like Italy, registering 4/6 times the estimated positive, compared to the real ones. A continuous stream of new information flows in every day regarding the COVID-19 statistics, and data to date suggest that 80% of the cases are mild or asymptomatic; 15% involve severe infection, requiring oxygen; and 5% indicate critical infection, requiring ventilation (WHO report 46).

Moreover, COVID-19 severe illness is more common in individuals having other health problems, particularly the elderly, such as those with cardiovascular disease, chronic lung disease, and hypertension [55]. People with diabetes are prominent among those high-risk categories that can have serious illness if they contract the virus [49]. In diabetic mellitus (DM), the MAPK cascade exerts a regulatory role in the pancreatic islet beta cell, in mediating the cellular responses to excessive generation of intracellular ROS (oxidative stress). Besides, novel pharmacological agents targeting the MAPK have the potential to improve the beta-cell function in diabetes [56]. Moreover, after insulin is absorbed by the endothelial barrier that mediates its actions in the muscle, heart, fat, and brain, the MAPK pathway enhances the ET-1 and PAI-1 expressions and the migration and proliferation of the contractile cells, which have proatherogenic actions. For conditions of insulin resistance or deficiency and in diabetes, there is a loss of the antiatherogenic actions of the insulin via the IRS/PI3K/Akt cascade, all of which causes an acceleration of atherosclerosis. By contrast, the activated MAPK pathway continues to exhibit proatherosclerotic actions [57]. The MAPK is also essential in mediating the pathogenesis of renal growth noticed in early DM. In a model of DM rats, the ERK1/2 MAPK levels and activity in the glomeruli are highly increased, also due to the loss of MAPK phosphatase-1, a dual specificity phosphatase that inactivates the MAPK [58], with a strong implication in the pathogenesis of DM nephropathy. It thus leads one to speculate that diabetic patients are more prone to COVID-19 infection and that subsequent death is due to the activation of the MAPK pathway upon viral activation and progeny [30, 59]. Moreover, the existing data on the downregulation of the MAPK pathway activity in response to the CQ and HCQ treatment should be taken into account, based mainly on the degree of severity of the COVID-19 disease in patients having additional health problems. The CQ application can control the glucose metabolism in DM patients as well [60, 61], as reviewed in [49]. Thus, the administration of the CQ in DM patients can boost the glucose metabolism and downregulate the MAPK pathway, which could contribute towards slowing down the viral activation and progeny.

## 7. Potential Cross Talk between MAPK Inhibitors, HCQ, and Derivatives in Viral Infection: Need for Basic Studies

Many patients worldwide have been treated already with HCQ during this pandemic situation [15]. The data presented here summarize that the HCQ treatments could affect at some level the MAPK pathway in regulating the different steps of viral replication, transport, translation, capsid assembly, and spread (Figures 1 and 2). Moreover, HCQ and the MAPK may also inversely control the inflammatory cytokine production and cellular apoptosis upon viral infection, thus being a rather clear target to fight against viral infection (Figure 3). Due to the initial discovery, nearly four decades ago, of the core elements of the MAPK pathway, considerable effort has been directed towards the development of the MAPK inhibitors which have shown promising clinical responses in cancer patients, both as single inhibitors or in combination [7]. This pandemic crisis forces us to consider high-throughput cellular and molecular approaches, to move from operational to scientifically driven pharmacovigilance. Common challenges, including the advancements in modern technology and data quality to strengthen translational science, are highly essential. Therefore, it is appealing to speculate that combinatorial approaches with low doses of both MAPK inhibitors and CQ, HCQ, or analogues are required to set up the optimal conditions, i.e., computing the combination index (CI) values or different drug combinations in all the experimental settings. Further, science that is more basic is necessary to have a clearer understanding of the mechanism of action of each individual drug, identify the vital molecular signaling mechanisms involved, and quantify the functional additional/synergistic effects. To achieve these results, both automatized platforms for cell viability/apoptosis assays and genome-wide siRNA screens and/or CRISPR-CAS9 technology are largely available. Of note, the genome-wide siRNA screens conducted on SARS-CoV [62] have identified MNK1 (MAP kinase interacting kinase 1) as one of the cellular factors that regulate the virus replication. Very recently, targeting the RAS/RAF/MEK and PI3K/AKT/mTOR downstream axes of GFR signaling was effective against SARS-CoV-2 replication [63]. The increase in detailed functional analyses of the host genes identified in the genome-wide screens will provide valid insights for the development of novel antiviral therapeutics.

## 8. Conclusions

Overall, the number of COVID-19 cases represents only the tip of the iceberg, since apparently 60-80% of the infected individuals are asymptomatic. The epidemiological classical measures of social distancing across the globe have reduced the overload in hospitals and intensive therapies, but the numbers continue to confirm that the infection curve is far from being under control and the risk of a new surge of cases over the next few months continues to lurk. Therefore, a good understanding of the role played by the MAPK cascade mechanisms in sustaining infection may be relevant during this pandemic. Moreover, any knowledge that can be added to the correlation between the MAPK cascade inhibitors and HCQ and HCQ analogues could pave the way to adopting new molecular approaches to tackle this severe disease, always remembering that a systematic strategy is the urgent requirement for the preclinical optimization of drugs to serve, especially, the population at risk.
